# Evidence of vertical transmission of Zika virus in field-collected eggs of *Aedes aegypti* in the Brazilian Amazon

**DOI:** 10.1371/journal.pntd.0006594

**Published:** 2018-07-16

**Authors:** Cristiano Fernandes da Costa, Arlesson Viana da Silva, Valdinete Alves do Nascimento, Victor Costa de Souza, Dana Cristina da Silva Monteiro, Wagner Cosme Morhy Terrazas, Ricardo Augusto dos Passos, Suzete Nascimento, José Bento Pereira Lima, Felipe Gomes Naveca

**Affiliations:** 1 Health Surveillance Foundation of Amazonas State FVS, Department of Environmental Surveillance, Manaus, Amazonas, Brazil; 2 Laboratório de Ecologia de Doenças Transmissíveis na Amazônia, Instituto Leônidas e Maria Deane – Fiocruz Amazônia, Manaus, Amazonas, Brazil; 3 Programa de Iniciação Científica, Instituto Leônidas e Maria Deane – Fiocruz Amazônia, Manaus, Amazonas, Brazil; 4 Programa de Pós-Graduação em Biologia Celular e Molecular, Instituto Oswaldo Cruz, Fiocruz, Rio de Janeiro, Brazil; 5 Programa de Pós-Graduação em Imunologia Básica e Aplicada, Universidade Federal do Amazonas, Manaus, Amazonas, Brazil; 6 Laboratory of Physiology and Control of Arthropod Vectors - Oswaldo Cruz Institute - FIOCRUZ, Rio de Janeiro, Brazil; 7 Programa de Pós-Graduação em Biologia da Interação Patógeno-Hospedeiro, Instituto Leônidas e Maria Deane – Fiocruz Amazônia, Manaus, Amazonas, Brazil; Independent Researcher, UNITED STATES

## Abstract

**Background:**

Arboviruses are viruses transmitted to humans and other animals by the bite of hematophagous arthropods. Infections caused by chikungunya virus (CHIKV), dengue virus (DENV), Zika virus (ZIKV), and the deadlier yellow fever virus (YFV) are current public health problems in several countries, mainly those located in tropical and subtropical regions. One of the main prevention strategies continues to be vector control, with the elimination of breeding sites and surveillance of infested areas. The use of ovitraps for *Aedes* mosquitos monitoring has already demonstrated promising results, and maybe be also useful for arboviral surveillance.

**Methods:**

This work aimed to detect natural vertical transmission of arboviruses in *Aedes aegypti* and *Aedes albopictus*. Mosquito egg collection was carried out using ovitraps in Itacoatiara, a mid-size city in Amazonas state, Brazil. Collected eggs were allowed to hatch and larvae were tested for CHIKV, DENV, and ZIKV RNA by RT-qPCR.

**Results:**

A total of 2,057 specimens (1,793 *Ae*. *aegypti* and 264 *Ae*. *albopictus*), in 154 larvae pools were processed. Results showed one positive pool for CHIKV and one positive pool for ZIKV. The active ZIKV infection was further confirmed by the detection of the negative-strand viral RNA and nucleotide sequencing which confirmed the Asian genotype. The Infection Rate per 1,000 mosquitoes tested was assessed by Maximum Likelihood Estimation (MLE) with 0.45 and 0.44 for CHIKV and ZIKV, respectively, and by Minimum Infection Rate (MIR) with 0.45 for both viruses.

**Conclusion:**

To our knowledge, this is the first detection of ZIKV in natural vertical transmission in the *Ae*. *aegypti*, a fact that may contribute to ZIKV maintenance in nature during epidemics periods. Furthermore, our results highlight that the use of ovitraps and the molecular detection of arbovirus may contribute to health surveillance, directing the efforts to more efficient transmission blockade.

## Author summary

The control of the vast majority of arbovirus infections relies on entomological measures to reduce mosquito infestation. Therefore, this study analyzed the use of ovitraps for arboviral surveillance in a mid-size city of the Amazonas state, Brazil. We found one larva pool infected with chikungunya virus, before the first human case confirmed in this municipality. Another pool was infected with Zika virus, demonstrating the first evidence that vertical transmission occurs in naturally infected *Aedes aegypti* mosquito populations.

## Introduction

The arboviruses transmitted by mosquitoes of the genus *Aedes*, like chikungunya virus (CHIKV), dengue virus (DENV), Zika virus (ZIKV), and yellow fever virus (YFV) have reached threatening numbers in the last years, with a huge impact on public health systems in several countries throughout the world [1–7]. Nevertheless, the detection and identification of circulating arboviruses are most often taken from human cases, mainly when an outbreak is already in place. With the fast-worldwide expansion of new emerging or reemerging arboviruses such as CHIKV, DENV, and ZIKV, the need to establish the role of each mosquito species in the spread of these pathogens is clear. This knowledge is fundamental to the implementation of effective surveillance and control measures against these vectors in order to avoid the early establishment of an arboviral epidemic [8].

The first report of natural infection of mosquitoes with ZIKV in Brazil was from *Aedes aegypti* adults collected in Rio de Janeiro [9] and this species was also considered as the primary vector during the epidemic. Indeed, the detection of ZIKV in *Ae*. *aegypti* mosquitos occurred soon after the emergence of ZIKV in this city.

A recent study showed the first description of *Ae*. *aegypti* infected with CHIKV ECSA genotype in Brazil [10]. These authors reinforce the role of this species as an important vector of CHIKV in urban areas of northeastern Brazil and emphasize the benefits of entomological surveillance programs for public health, regarding the immediate implementation of diseases prevention.

A study conducted in the Amazonas State, Brazil, demonstrated that the entomological surveillance using ovitraps could be successfully used to monitor the different DENV serotypes circulating in the municipalities of the interior of the state. Moreover, this study suggests that the use of arboviral monitoring strategies in routine surveillance helps for early detection of virus circulation before outbreaks, contributing to more efficient and effective control measures [11].

The continuous monitoring of *Ae*. *aegypti* infestation, associated with the early detection of arbovirus circulation, may contribute to the development of epidemic prediction models for diseases transmitted by this vector. Besides, vertical transmission, even at a low rate, contributes to the preservation of an arbovirus in nature, without a well-known cycle involving invertebrates and vertebrates, and may be of paramount importance in endemic areas as an alternative arboviral maintenance mechanism [12,13].

In the present study, we monitored and detected the natural vertical transmission of arboviruses in a mid-size city of the Amazonas State, Brazil, during the emergence of ZIKV in 2015–2016.

## Materials and methods

### Ethics statement

The collection of *Aedes* eggs doesn't require special permission in Brazil. All the house owners agreed with and allowed the installation of ovitraps in their properties.

### Study area and mosquito egg collections

The study area was the city of Itacoatiara in the Amazonas State, located in Northern Brazil. Amazonas is the largest state of the Brazilian federation with 1,559,161 km^2^ and an estimated population of 4,063,614 inhabitants (2.23 inhabitants/km^2^). Amazonas has international borders with Venezuela, Colombia, and Peru, and domestic borders with the states of Roraima, Pará, Mato Grosso, Rondônia, and Acre. Itacoatiara (latitude 03° 08' 35" S; longitude 58° 26' 39" W) is located 189km from the capital Manaus and is the third most populous municipality in the Amazonas with an area of 8,891.9 km^2^ and a population estimated at 100,000 inhabitants (9.77 inhabitants/km^2^). The predominant climate is equatorial (tropical monsoon), characterized by high temperatures and a significant rainfall over ≥ 2500 mm per year, but with a dry season also known as Amazonian winter. An important fluvial harbor is located in Itacoatiara for the transport of inhabitants and agricultural products [14].

Our group has been using ovitraps to monitor *Aedes* spp. infestation. The ovitraps consist of a dark plastic container with a capacity of 700 mL containing 300 mL of a 0.04% brewer's yeast solution as an attractant for mosquito females, and an oviposition substrate on which the eggs are laid (Eucatex, Brazil) with the rough part facing the inner area of the trap for oviposition. On each monitoring cycle, a total of 100 ovitraps were installed around selected houses in the city, with a weekly exchange of the pallets. Ovitraps distribution was equidistant on every 200 meters, covering the entire inhabited area of the city.

All of the properties where the ovitraps were installed were georeferenced with the use of a GPS device (Garmin Ltd, USA#MP62SC) with UTM—SIRGAS 2000 projection. The coordinates were inserted into a geographic information system (GIS) in the software QGIS 2.16.2, where each property had its spatial location identified as a point (attribute) in a layer (shape) of the city.

Initially, collected pallets were sent to the Itacoatiara entomology laboratory, placed to dry and analyzed under a stereoscopic magnifying glass for *Aedes* spp. eggs count. Once every 2 months, the positive pallets were sent to the entomology laboratory of the Fundação de Vigilância em Saúde do Amazonas (FVS-AM), where the pallets were individually immersed in glass flasks containing 200 mL of dechlorinated water for egg hatching.

The larvae were raised until the third stage when species were identified and separated into pools of a maximum of 30 specimens, placed in cryotubes, and sent frozen to Instituto Leônidas e Maria Deane (ILMD)–Fiocruz Amazônia, where they were kept in a -80°C freezer, until molecular analysis for arboviral RNA detection.

### Total RNA extraction

Firstly, each larvae pool was spiked with the *Escherichia coli bacteriophage MS2* (ATCC 15597-B1) to be used as an internal positive control (IPC) using the same conditions previously described [15]. All pools were individually disrupted in 2 mL microtubes containing a 5 mm stainless-steel bead and 250 μL of TRIzol Reagent (Invitrogen, USA, #15596026) with the aid of the TissueLyser LT bead mill (Qiagen, Germany, #85600), 50Hz for 5 minutes. The reservoir containing the microtubes was frozen and kept in an ice bath during the process. Posteriorly, the homogenized pools were clarified by centrifugation, and the supernatant was added with 750 μL of Trizol. Therefore, RNA extraction followed the manufacturer’s recommendations. The RNA pellet was resuspended in 40 μL of nuclease-free water and evaluated for quantity and quality with the BioDrop DUO UV spectrophotometer (BioDrop Ltd, United Kingdom, #80-3006-61). A total of 2.5 μL microliters of the extracted RNA was used for the RT-qPCR assays. The remaining volume was stored at -80°C for further analyses.

### Viral RNA detection by RT-qPCR

All samples were evaluated for the detection of three arboviral RNAs by RT-qPCR in a StepOnePlus Real-Time PCR System (Applied Biosystems, USA, #4376598) located at the Real-Time PCR Platform of ILMD. The protocols used for the detection of each virus were previously published: DENV [16]; CHIKV [17]; and ZIKV [18], but we conducted the assays with some modifications. All probes were used at a final concentration of 0.1μM, whereas all primers were used at a final concentration of 0.3μM. All reactions were performed with TaqMan Fast Virus 1-Step Master Mix (Applied Biosystems, USA, #4444432), following manufacturer’s recommendations, except for the number of cycles that was increased to 45. For each lot of analyzed samples, three blank reactions (nuclease-free water as the template) and external positive controls (RNA extracted from viral culture) were included. The number of viral copies in each positive sample was estimated by RT-qPCR using absolute quantification by the standard curve method and reported as viral RNA copies/μL (of the eluted RNA).

### Evaluation of ZIKV productive infection by RT-qPCR

With the objective to evaluate the ZIKV replication in naturally infected larvae, we conducted the same RT-qPCR assay used for detection of ZIKV RNA, but in a two-step protocol targeting the negative-strand RNA. Firstly, two different cDNA assays were made: I) with only the reverse primer (which hybridizes to positive-strand RNA) and II) with only the forward primer (which hybridizes to negative-strand RNA). All cDNAs were made with SuperScript IV Reverse Transcriptase (Invitrogen, Carlsbad, CA, #18090050), according to the manufacturer’s recommendations.

Subsequently, a qPCR assay was conducted with TaqMan Fast Advanced Master Mix (Applied Biosystems, USA, #4444558), following manufacturer’s recommendations, except for the number of cycles that was increased to 45. Three different master mixes we used: I) with only the reverse primer; II) with only the forward primer and III) with both reverse and forward primers.

### Conventional RT-PCR and nucleotide sequencing

The positive pools were submitted to a conventional RT-PCR amplification protocol using the primers ZIKA_ASIAN_FNF1 (5’–CCGCGCCATCTGGTATATGT– 3’) and ZIKA_ASIAN_FNR (5’–CTCCACTGACTGCCATTCGT– 3’) designed to target the NS5 coding region of Asian ZIKV lineages. For the CHIKV sample, we used a protocol already described for alphaviruses amplification [19].

Thereafter, amplicons were precipitated with PEG-8000 and submitted to the nucleotide sequencing reaction with BigDye Terminator v3.1 Cycle Sequencing Kit. Capillary electrophoresis was conducted in an ABI3130 sequencer located at the genomics platform of ILMD, Fiocruz Amazônia. The final FASTA sequences were initially submitted to BLAST analysis [20] and further evaluated by a web-based Dengue, Zika and Chikungunya Subtyping Tool (Version 1.0), freely available at http://bioafrica.mrc.ac.za/rega-genotype/html/index.html.

### Arbovirus infection rates in larvae

The virus infection rate (IR) was calculated with PooledInfRate, version 4.0 by Biggerstaff, a Microsoft Excel add-in that computes the IR using data from pooled samples (even with different pool sizes) by both Minimum Infection Rate (MIR) and Maximum Likelihood Estimation (MLE) methods. Freely available at https://www.cdc.gov/westnile/resourcepages/mosqsurvsoft.html.

## Results

Between January and April 2016, 154 larvae pools containing 2,057 specimens (1,793 *Ae*. *aegypti* and 264 *Ae*. *albopictus*) were analyzed for CHIKV, DENV, and ZIKV RNA. By using the RNA extraction method described under Material and Methods we were able to obtain high quality RNA for most samples (260/280 median value: 1.96; IQR: 1.87–2.01) and all IPC reactions were positive (Ct median value: 30.9; IQR: 30.0–31.4).

**Fig 1:**
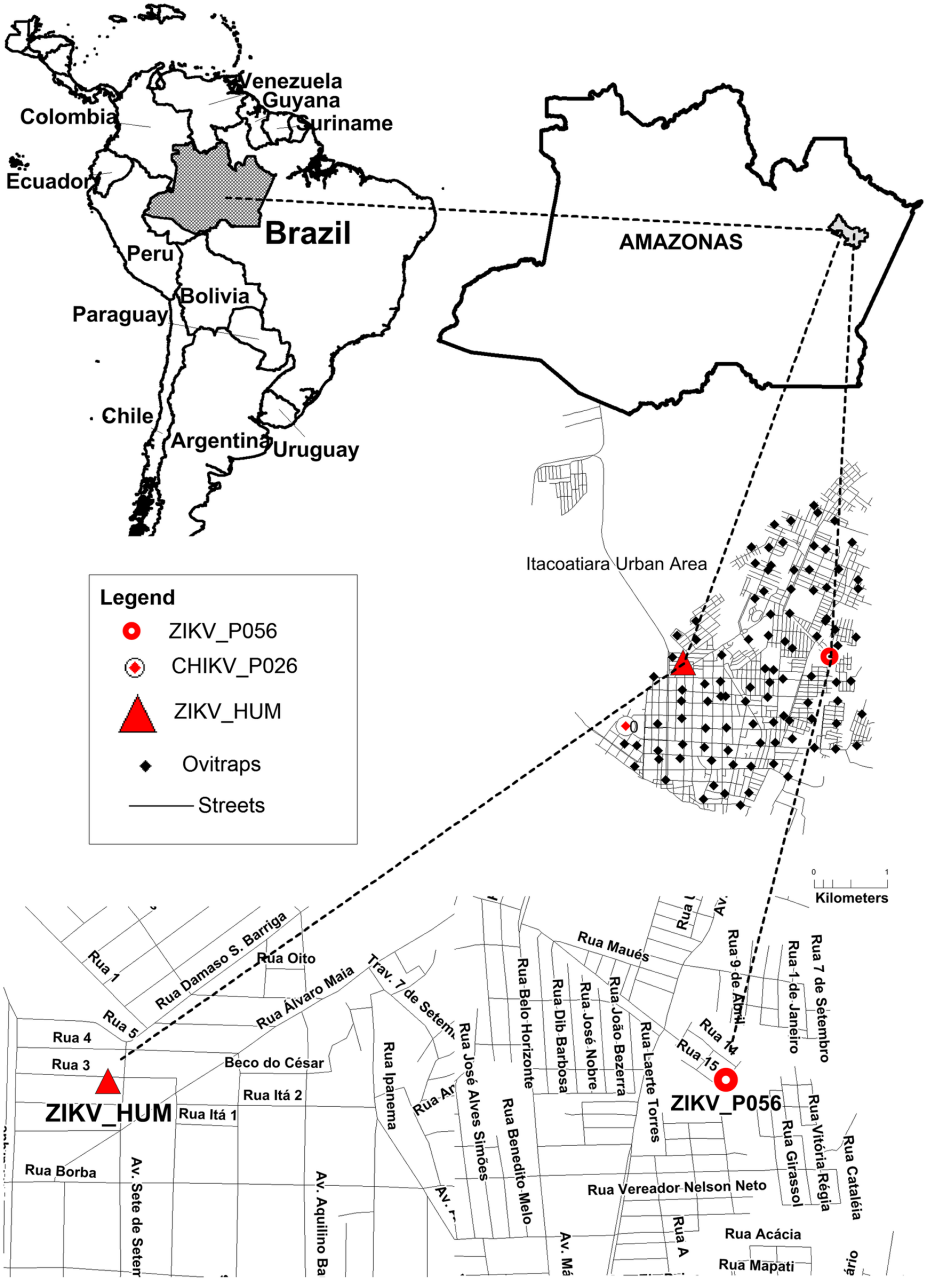
Map of the Itacoatiara city in the context of the Amazonas State, Brazil. The location of each ovitrap is represented by a black diamond. The red circles represent the location of positive arbovirus ovitraps. The red triangle represents the human Zika case.

One pool containing only one larvae, obtained from the trap P056/ITA, collected at 15-Feb-2016, was positive to ZIKV (47 viral RNA copies/μL, approximately 1.8 x 10^3^ ZIKV RNA copies in the infected larvae). Another pool containing three larvae, obtained from the trap P026/ITA, collected at 25-Feb-2016, was positive to CHIKV (2 viral RNA copies/μL) Fig 1.

The positive ZIKV pool was further evaluated for productive infection by the specific detection of the negative-strand RNA. We detected amplification for both cDNAs, derived from the positive or negative-strand RNA, with a positive-to-negative strand RNA ratio of approximately 2:1, represented by the difference of one Ct (Table 1).

**Table 1:** Strand-specific amplification of ZIKV RNA from infected *Aedes aegypti* larvae.

cDNA	qPCR		
	ENV-F^c^	ENV-R^d^	ENV-F+R^e^
ENV-R^a^	32.5*	Negative	32.2*
ENV-F^b^	Negative	33.4*	33.3*

Two different cDNA were produced with ENV-Reverse^a^ primer or ENV-Forward^b^ primer and RNA extracted from the infected larvae pool. The qPCR master mixes were made only with forward primer (ENV-F^c^); only with reverse primer (ENV-R^d^) or with both primers (ENV-F+R^e^).

* Median Ct values from qPCR experiments in triplicate. Amplification of the negative-strand RNA is highlighted in bold.

Subsequently, conventional RT-PCR amplification was performed on both positive pools as described in Material and Methods, but unfortunately, only the Zika sample could be amplified. The final ZIKV sequence corresponds to a fragment of 450bp that was deposited in GenBank under the accession number MG279550. The analysis of the viral genotyping confirmed the Asian ZIKV lineage.

The virus IR per 1,000 mosquitoes tested was calculated for CHIKV and ZIKV based in the total of pools and individuals tested. The CHIKV IR was 0.45 for both MIR and MLE methods (MIR: lower Limit = 0.00; upper limit = 1.32 and MLE: lower Limit = 0.03; upper limit = 2.15). The ZIKV IR was 0.45 for MIR (MIR: lower Limit = 0.00; upper limit = 1.32) and 0.44 for MLE (MLE: lower Limit = 0.03; upper limit = 2.15).

## Discussion

The first report about the detection of *Ae*. *aegypti* in Manaus, the capital of Amazonas State, was in November 1996 and for *Ae*. *albopictus* in September 1997. Since then, *Aedes* spp. mosquitos began to be found in other municipalities of the interior of the Amazonas State, and successive *Aedes*-related arboviral infections have been reported [21–25].

Between 2015 and 2016, a total of 878 suspected cases of CHIKV and 4,485 cases of ZIKV were reported in the Amazonas State [26,27]. Preventive and reactive measures regarding the vector control were carried out by the health authorities under the coordination of FVS-AM, to decrease *Aedes* infestation in different municipalities. In addition to the routine actions, ovitraps were installed with the purpose of directing the field efforts more efficiently.

The primary aim of our study was to evaluate natural arboviruses vertical transmission in the field. Since this study was conducted under circumstances where the cold-chain could not be guaranteed, we decided to use a study design that favored the molecular detection, protecting viral RNA as soon as possible. Therefore, we decided to disrupt the *Aedes* larvae directly in Trizol reagent, which consists of a solution of phenol and guanidinium isothiocyanate that concurrently solubilizes biological material and immediately inactivates RNases [28], but also inactivates viral particles, preventing the possibility of viral isolation.

The present study confirms other studies demonstrating natural vertical transmission of CHIKV [29–31], in a municipality with no human case previously confirmed. It is important to emphasize that most acute febrile cases in Brazil are diagnosed by clinical examination, without specific laboratory confirmation, especially during an ongoing outbreak. At the time we collected our samples, a Zika outbreak was already established, which could compromise the diagnosis of chikungunya cases. Although the CHIKV positive sample was amplified in duplicate in the probe-based RT-qPCR assays, we were unable to amplify this sample using conventional RT-PCR, preventing its sequencing. RT-qPCR would have been more sensitive compared to conventional RT-PCR, particularly for samples with low viral load. Another important point is that the sequence variation at the primers sites that may also decrease the efficiency of nucleic acid amplification methods.

In an experimental study of vertical ZIKV infection, a total of 69 pools of *Ae*. *aegypti* adult mosquitos (F1) were tested, and six were positive in an indirect immunofluorescent antibody assay [32]. While other studies have demonstrated the natural vertical transmission of DENV [11,33–37], the detection of ZIKV in naturally infected larvae had not yet been described.

We report the first detection of Zika virus vertical transmission in an *Ae*. *aegypti* larvae under the natural conditions found in the field. Therefore, we evaluated if there was a productive infection in the *Ae*. *aegypti* larvae by strand-specific amplification of viral RNA. ZIKV is a positive-sense, single-stranded RNA virus that belongs to the *Flaviviridae* family. During flaviviruses replication, a complementary negative-strand RNA is produced, which is used as a template for synthesizing new positive-strand RNA copies. Viral replication progresses asymmetrically, producing more positive-strand than negative-strand RNA. The positive-strand RNA molecules are packaged into the virions, acts as templates for viral protein production and promotes evasion of innate cell response [38,39]. Therefore, the specific detection of negative-strand flavivirus RNAs is an indicator of active viral replication, and different studies have been using this approach [40–43].

In this study, ZIKV cDNA was produced with the reverse primer, which hybridizes with the positive-strand RNA, e.g., the genomic RNA found into the virion particle and also detected during viral replication, or with the forward primer, which hybridizes with the negative-strand RNA, only found during replication. The qPCR results clearly showed that both cDNAs were amplified in a positive-to-negative strand RNA ratio of approximately 2:1.

To the best of our knowledge, there is only one plausible explanation that could explain the detection of ZIKV RNA in the larvae, besides a canonical viral infection. Recently, different studies showed that naked viral RNA from hepatitis C virus (HCV), as well as from human pegivirus, two members of the *Flaviviridae* family (genus *Hepacivirus* and *Pegivirus*, respectively), may spread infection in exosomes vesicles [44,45]. Importantly, these studies showed the phenomenon *in vitro*, and further investigations are required to prove if similar events occur during ZIKV infection *in vivo*. Furthermore, the same studies also showed that, regardless of the way of viral RNA release in the cytoplasm of newly “infected” cells, productive viral RNA replication was observed, leading to the release of infectious particles. Given this, although our study design does not allow us to assert if ZIKV RNA reaches larvae cells by a classic route of viral infection, the specific detection of the negative strand RNA provides substantial evidence that active viral replication has occurred in *Ae*. *aegypti* naturally infected larvae.

We detected arboviral RNA in larvae, which does not necessary means that larvae would become infected adults due to possible transstadial loss of infection during development to adulthood. On the other hand, if some of the infected larvae achieve maturity still infected, they will be readily able to transmit ZIKV to other mosquitos by venereal transmission [46] or, in the case of females, to human hosts. Thus, this phenomenon may contribute to the epidemic potential of this arbovirus because mosquitoes that emerge as virus-infected adults will have more opportunity to transmit virus than mosquitos that become infected after blood meal in an infected vertebrate. Further studies are necessary to evaluate all variables contributing to maintaining a virus circulating in a specific area until the number of new susceptible human subjects raise, by immigration or births, sufficiently to support a new epidemic cycle.

According to previous work, the rapid detection of arbovirus in specimens collected in the field may contribute to the effectiveness of vector control measures, decreasing the viral transmission among the human population [47]. Altogether, the results showed in the present manuscript strengthen the importance of continuous monitoring of arboviral infections in both mosquitoes, as well as in human hosts, before the establishment of a new outbreak.
